# Agave associated crinivirus A: a novel monopartite crinivirus homolog isolated from agave

**DOI:** 10.1007/s00705-026-06580-x

**Published:** 2026-03-20

**Authors:** Kristian A. Stevens, Juliana Osse de Souza, Haoran Li, Ashrafou Ouro-Djobo, Olufemi J. Alabi, Maher Al Rwahnih

**Affiliations:** 1 Foundation Plant Services, University of California-Davis, Davis, CA 95616 USA; 2 Department of Computer Science and Evolution and Ecology, University of California-Davis, Davis, CA 95616 USA; 3 Department of Plant Pathology, University of California-Davis, Davis, CA 95616 USA; 4 Department of Plant Pathology & Microbiology, Texas A&M AgriLife Research and Extension Center, Weslaco, TX 78596 USA; 5 Department of Plant Protection, School of Agriculture, The University of Jordan, Amman, Jordan

## Abstract

We describe the complete genome of the first monopartite and putative member of the genus *Crinivirus* which we propose naming agave associated crinivirus A (AaCA). AaCA was identified by high-throughput sequencing in an *Agave tequilana* leaf sample during a routine metagenomic screening of Agave plants from California. The 16,161 bp genome contains the protein hallmarks of the family *Closteroviridae*, the HSP70h and the three coat protein homologs (CPh, CP, CPm), along with the open reading frames (ORFs) unique to criniviruses. Two ORFs downstream of the CPm are unique to AaCA. The monopartite nature of the genome was verified by PCR and Sanger sequencing. Phylogenetic analysis of the HSP70h gene clusters AaCA basally with existing criniviruses.

**Supplementary Information:**

The online version contains supplementary material available at 10.1007/s00705-026-06580-x.

The genus *Crinivirus* is one of the four genera that currently comprise the family *Closteroviridae* which exclusively infect plants, having evolved to replicate and move within the plant’s phloem tissue. They primarily infect dicots, including many economically important agricultural crops, and as a result have been agents of significant agricultural losses [1]. While multiple vectors are known to transmit members of the family *Closteroviridae* [2], species of the taxonomically distinct genus *Crinivirus* are exclusively transmitted by whiteflies in the genera *Trialeurodes* and *Bemisia* [1]. Crinivirus infections are therefore associated with tropical and subtropical climates where populations of whiteflies are prevalent [3].

The first member of the genus identified was beet pseudo-yellows virus (BPYV) [4]. It can infect beets, lettuce, and cucumbers and causes chlorosis and reduced vigor [3]. At the time of writing, there are now 14 member species of the genus recognized by the International Committee of Taxonomy on Viruses (ICTV) and 11 additional taxonomically related viruses are awaiting ratification [5]. Several criniviruses are economically significant due to their impact on crop production worldwide. Lettuce infectious yellows virus (LIYV) causes yellowing and stunting and is associated with major losses in lettuce and melon crops [6]. Sweet potato chlorotic stunt virus (SPCSV) causes yield reduction in sweet potato, especially in mixed infections with other viruses, including the potyvirus sweet potato feathery mottle virus (SPFMV) [7]. Tomato chlorosis virus (ToCV) is widespread in tomato-producing regions and leads to chlorosis and reduced fruit quality [3]. Cucurbit yellow stunting disorder virus (CYSDV) causes yellowing and stunting and is a major threat to cucurbit crops [3].

Criniviruses are also unique among the family *Closteroviridae* for their multi-partite genomic architecture. All known criniviruses are bipartite except for potato yellow vein virus (PYVV) [8], which has a tripartite structure [9]. The bipartite genome is divided into two linear, positive-sense, single-stranded RNAs totaling 14.9–17.9 kb [9, 10]. Furthermore, criniviruses exhibit bipartite encapsulation into filamentous, non-enveloped particles, where each genomic RNA is separately encapsidated into its own virion, and both particles must be present together for a successful infection. The major coat protein (CP) forms the majority of the virion, and the minor coat protein (CPm) forms the tail end and is crucial for whitefly transmission [10]. Additional proteins like heat shock protein 70 homolog (HSP70h) and a p59, also referred to as the CPh, are also part of the virion structure and contribute to its assembly and stability.

In 2022, ten *Agave tequilana* plant samples were collected for virus screening from a single cultivated area near Fresno California comprising approximately 25000 row cropped plants from diverse nurseries. Symptoms were not documented for this routine screening. In one leaf sample, a novel virus was detected using high-throughput sequencing (HTS) at Foundation Plant Services (FPS), University of California, Davis. For increased coverage and improved contiguity, the final HTS analysis of this sample utilized three replicate total RNA extracts from the same biological leaf sample obtained using a MagMAX Plant RNA Isolation Kit (Thermo Fisher Scientific, Waltham, MA), following the manufacturer’s protocol. The RNA was first depleted of its ribosomal RNA content and then used for complementary DNA (cDNA) library preparation using a TruSeq Stranded Total RNA with Ribo-Zero Plant Kit (Illumina, Inc., San Diego, CA). The first replicate cDNA library was single-end sequenced on an Illumina NextSeq 500 platform, generating approximately 18.7 million reads, each 75 nucleotides (nt) in length. Subsequently, two replicate libraries were sequenced on an Illumina NextSeq 2000 platform, generating approximately 22.0 and 27.0 million read pairs of 100 nucleotides in length. Trimming, host-screening, *de novo* assembly, and BLASTX analysis of the HTS reads were done as described in [11]. From a combined denovo assembly, the longest sequence (15,262 bp) was selected as the basis for the rapid amplification of cDNA ends (RACE) assays to extend and verify the genome ends. RACE assays were performed using a SMARTer RACE 5′/3′ Kit (Takara Bio USA, Inc.) according to the manufacturer’s recommended protocols. This step extended the genome in the 5’ and 3’ directions by 28 and 871 nucleotides, respectively. Additional PCR validation was performed to verify the non-chimeric nature of the assembly (see below).

The full genome of the novel virus, tentatively named “Agave associated Crinivirus A” (AaCA; GenBank accession number PX549190), is 16,161 nt long. The most remarkable aspect of the genome organization of AaCA is its novel and ancestral monopartite structure. The single RNA genome contains all the major open reading frames (ORFs) associated with both *Closteroviridae* and *Crinivirus* membership (Fig. 1, Table 1). Specifically, the HSP70h and the three coat protein homologs (CPh, CP, CPm) unique to the family *Closteroviridae* are present. HSP70h of AaCA shares up to 45% identity with other criniviruses. The major CP, shares 29–32% protein identity with other criniviruses, while the CPm shares 25–35% protein identity with other criniviruses. A CPh is also present and similar to the other virion-associated CPh/p59 proteins, sharing up to 27% protein identity. The RNA-dependent RNA polymerase (RdRp) is the most conserved ORF, sharing up to 56% protein identity with other members. ORFs unique to criniviruses are also present in the AaCA genome, including a p10 between the CPh and CP proteins and a p26 protein which is the very last ORF in the genome organization. The p26 ORF does not exhibit sequence similarity to other viral proteins. This is not particularly remarkable; for example, the p26 of LIYV has no homology to other viral proteins even though it plays an important role in host infection [12]. Finally, at the 3’ end between the CPm and the terminal p26 there are two additional novel ORFs. A p47 ORF, with partial protein homology to the CPm and a p24 with no sequence homology. The CPm/p47 sequence similarity is observed in the translated dot plot as an ancestral tandem duplication covering 49% of the p47 at 38% protein identity (see supplement).

**Fig. 1:**
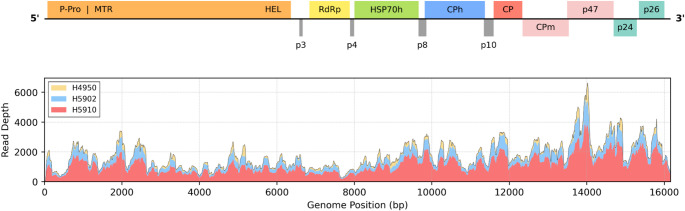
Genome organization with the read depth from three HTS runs of the infected sample. Notably, read depth was contiguous across the entire monopartite genome in all samples. Figure created using VirPlot (https://github.com/ucdavis/VirPlot)

**Table 1:** Amino acid identities between main proteins of AaCA and other criniviruses

Virus	ORF1	RdRP	HSP70h	CPh	CP	CPm	P47
Lettuce infectious yellows virus	34.3%	52.4%	40.8%	27.2%			
Tomato infectious chlorosis virus	35.8%	50.4%	40.8%	26.2%		27.7%	
Plant associated closterovirus 2	35.3%	53.3%	45.0%	23.4%		24.7%	
Sweet potato chlorotic stunt virus	36.0%	52.7%	43.6%	26.4%	31.6%	31.0%	28.1%
Potato yellow vein virus	34.4%	54.1%	42.4%	23.7%	31.7%	24.1%	27.7%
Arracacha latent virus C	34.7%	55.4%	41.7%	23.2%	30.8%	24.2%	
Blackberry yellow vein-associated virus	34.4%	54.1%	42.8%	22.2%	29.4%	32.6%	
Mulberry crinivirus	37.0%	51.8%	41.5%	24.8%	31.7%		
Strawberry pallidosis-associated virus	35.5%	53.9%	40.9%	21.8%	30.9%		
Diodia vein chlorosis virus	33.6%	54.9%	42.0%	24.0%	29.7%		23.7%
Cucumber yellows virus	31.8%	54.3%	41.7%	23.6%	30.6%		
Beet pseudo-yellows virus	33.6%	56.1%	41.7%	24.0%	30.6%		
Yam virus 1	31.9%	51.0%	41.8%	25.5%	29.5%	34.7%	
Cucurbit yellow stunting disorder virus	33.0%	50.7%	43.6%	26.2%	30.7%	26.8%	32.3%
Tetterwort vein chlorosis virus	33.3%	52.0%	41.7%	26.6%	31.0%		
Bean yellow disorder virus	32.5%	51.3%	40.7%	25.1%		34.7%	
Lettuce chlorosis virus	32.8%	51.3%	40.6%	26.5%			28.9%

A phylogenetic analysis was conducted using amino acid sequences of HSP70h of representative members of the family *Closteroviridae*, obtained from the NCBI GenBank. Multiple sequence alignments were generated with MAFFT v7.526 [13]. Subsequent phylogenetic reconstruction was performed with IQ-TREE v3.0.1 [14] using the maximum-likelihood framework, applying 1,000 bootstrap replicates with a minimum branch support of 80. The resulting tree was visualized and annotated with iTOL v7.2 [15]. Notably, AaCA clusters basally with other members of the *Crinivirus* genus and is separated from the genus *Velarivirus*. This basal clustering with the crinivirus clade was also observed for reconstructions performed using the RdRP, and CPm amino acid sequences (see Supplement). This is consistent with a hypothesis of the ancestral nature of a monopartite *Closteroviridae* genome, with other members of *Crinivirus* having later developed bi- and tri-partite genomic architectures (Fig. 2).

**Fig. 2:**
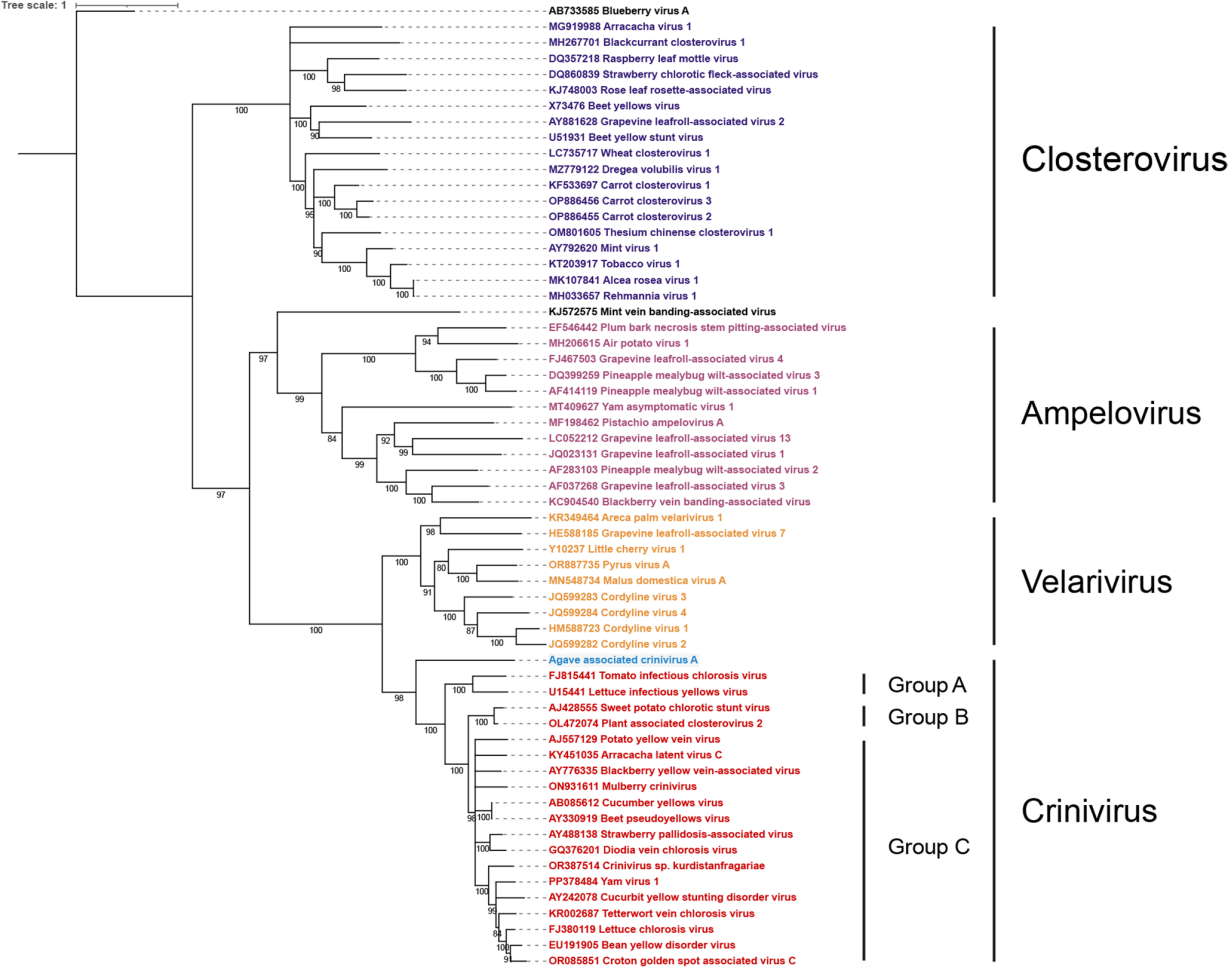
The phylogenetic relationship of agave-associated crinivirus A with other members of the family *Closteroviridae* using HSP70h amino acid sequences. Notably, AaCA clusters basally with other members of the Crinivirus genus and is separated from the genus Velarivirus

To confirm the presence of AaCA in the sample and validate its monopartite nature, PCR primers AaCA-1_6162F (5’-TTGTGAGGTTGAGTCGCTATG-3’)/AaCA-1_8419R (5’-GGCGGGAACGCTGATATTTA-3’) were designed to amplify a 2.3 kb genomic region beginning from the RdRP and stretching into the HSP70h that in other criniviruses would normally be found on RNA1 and RNA2, respectively. Using these primers, we were able to successfully amplify and Sanger sequence the region as a contiguous sequence fragment, positively confirming the presence of the virus in the sample and its monopartite genomic architecture.

In summary, we report the complete genome sequence of a novel crinivirus homolog putatively named agave associated crinivirus A (AaCA) isolated from *Agave tequilana*. The species name *Crinivirus agave* is proposed for AaCA. It is the first crinivirus homolog to be reported with a monopartite genome and clusters basally in a clade with other criniviruses. Further work will be needed to determine if AaCA is vectored by whiteflies like other criniviruses, though it is notable that the native and cultivated ranges of agave overlap significantly with the range of the whitefly vector. Further studies will also be needed to determine the host range of AaCA, its genetic diversity and distribution among wild and cultivated agave, and its potential contributions to disease symptoms.

## Supplementary Information

Below is the link to the electronic supplementary material.

Supplementary Material 1

Supplementary Material 2
