# Supplementary material for: Agave associated crinivirus A: a novel monopartite crinivirus homolog isolated from agave
Source: Arch Virol. 2026 Mar 20;171(4):138. doi: 10.1007/s00705-026-06580-x (PMC13004744; doi:10.1007/s00705-026-06580-x)

**SUPPLEMENTARY FIGURES**

Figure S1 Phylogenetic tree constructed from the duplicated CPm is very similar to the one constructed from the HSP70 and also puts the AaCA lineage at the base of the Crinivirus clade.


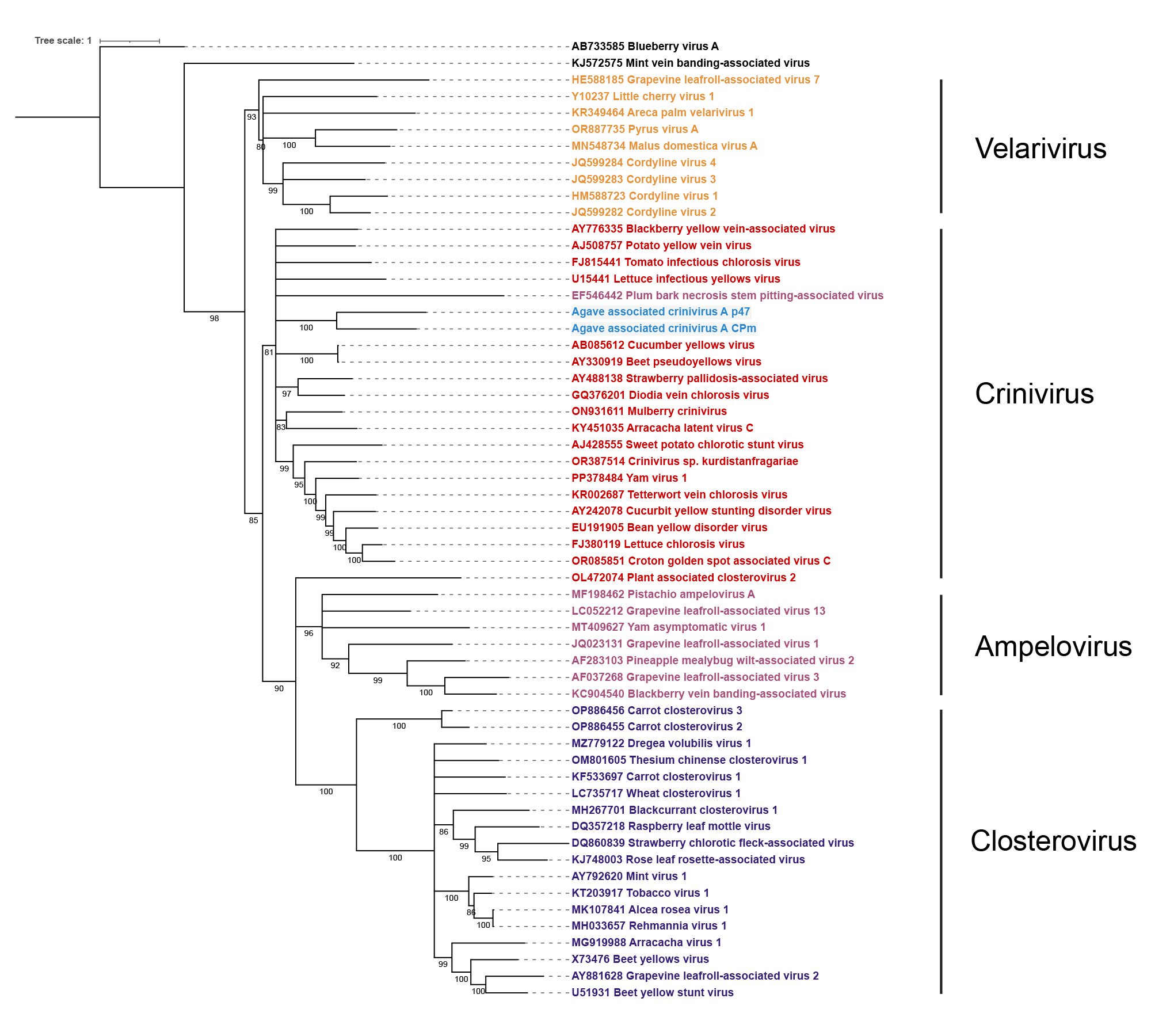


Figure S2 Phylogenetic tree constructed from the RdRp is very similar to the one constructed from the HSP70 and also puts the AaCA lineage at the base of the Crinivirus clade.


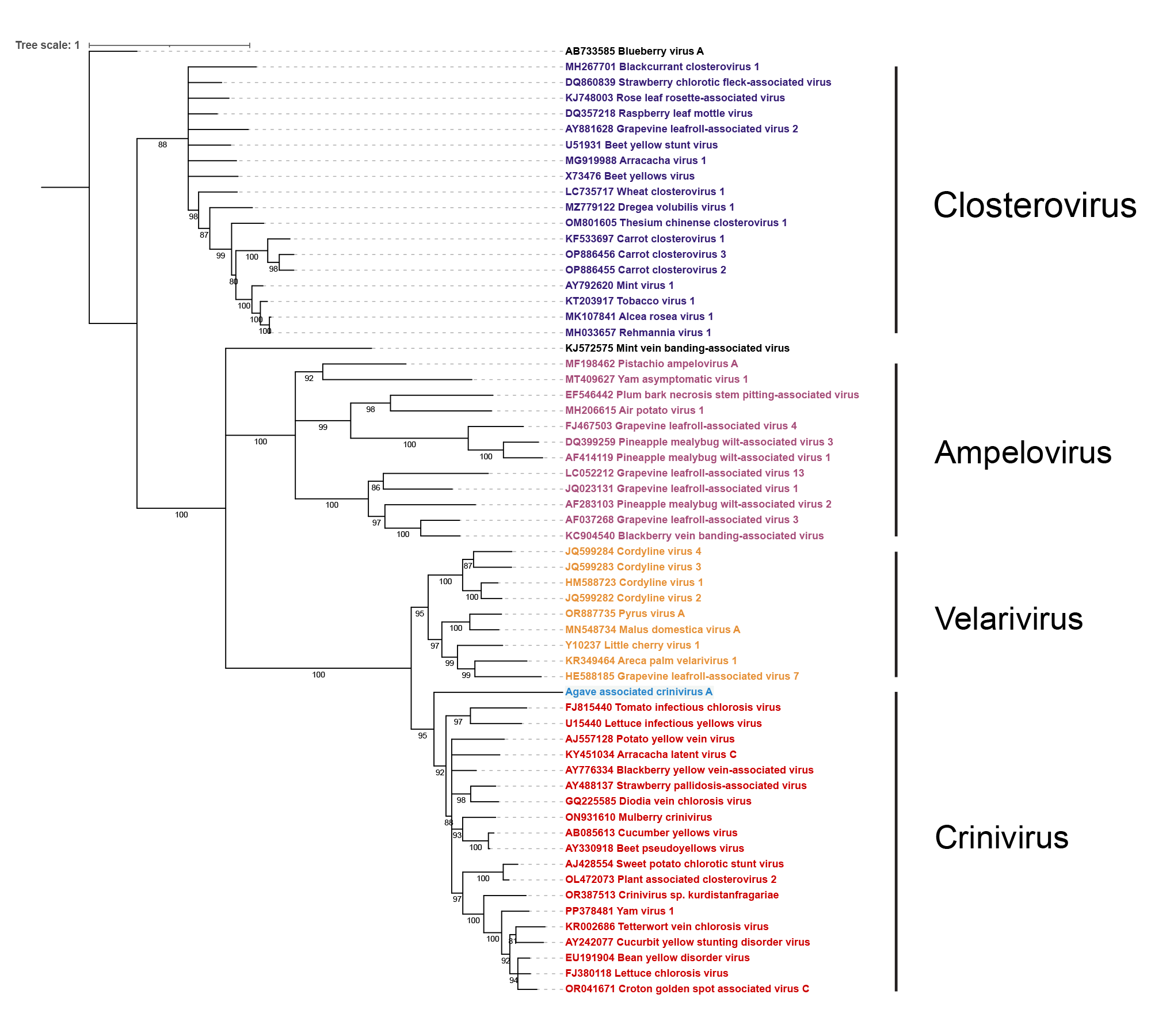


Figure S3 Phylogenetic tree constructed from the CP is very similar to the one constructed from the HSP70 and clusters the AaCA lineage adjacent to LIYV.


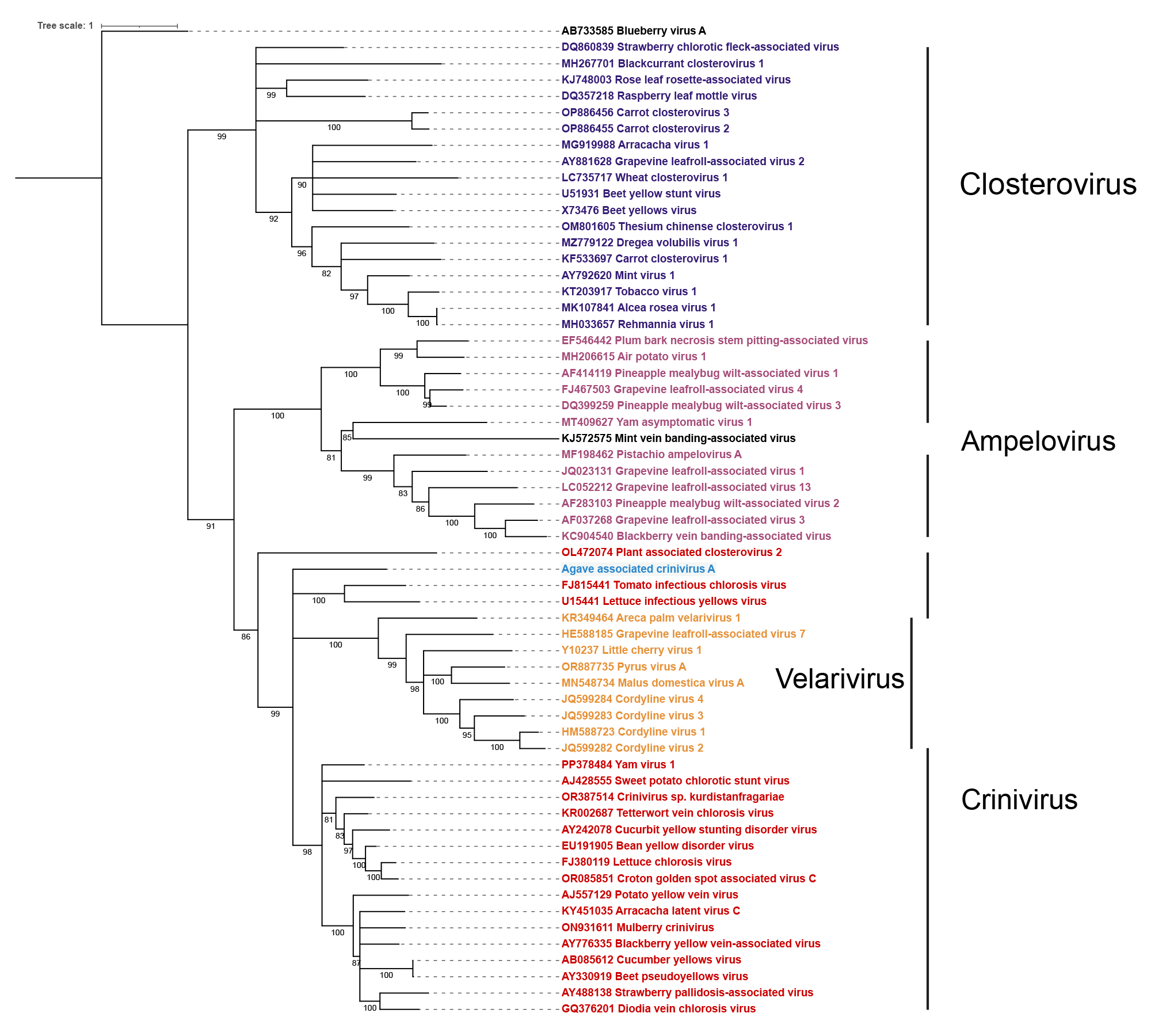


Figure S4 Translated nucleotide dotplot showing the repeat structure at the 3' end of the virus. The parallel lines denote the repeat that gave rise to the paralogous CPm and p47 open reading frames.


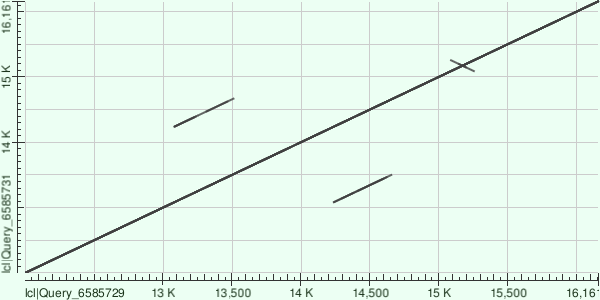

Supplement: Supplementary file 2 — Supplementary Material 2 [file 705_2026_6580_MOESM2_ESM.docx]
